# The role of local impedance drop in the acute lesion efficacy during pulmonary vein isolation performed with a new contact force sensing catheter—A pilot study

**DOI:** 10.1371/journal.pone.0257050

**Published:** 2021-09-16

**Authors:** Nándor Szegedi, Zoltán Salló, Péter Perge, Katalin Piros, Vivien Klaudia Nagy, István Osztheimer, Béla Merkely, László Gellér

**Affiliations:** Heart and Vascular Center, Semmelweis University, Budapest, Hungary; Ohio State University, UNITED STATES

## Abstract

**Introduction:**

Our pilot study aimed to evaluate the role of local impedance drop in lesion formation during pulmonary vein isolation with a novel contact force sensing ablation catheter that records local impedance as well and to find a local impedance cut-off value that predicts successful lesion formation.

**Materials and methods:**

After completing point-by-point radiofrequency pulmonary vein isolation, the success of the applications was evaluated by pacing along the ablation line at 10 mA, 2 ms pulse width. Lesions were considered successful if loss of local capture was achieved.

**Results:**

Out of 645 applications, 561 were successful and 84 were unsuccessful. Compared to the unsuccessful ablation points, the successful applications were shorter (p = 0.0429) and had a larger local impedance drop (p<0.0001). There was no difference between successful and unsuccessful applications in terms of mean contact force (p = 0.8571), force-time integral (p = 0.0699) and contact force range (p = 0.0519). The optimal cut-point for the local impedance drop indicating successful lesion formation was 21.80 Ohms on the anterior wall [AUC = 0.80 (0.75–0.86), p<0.0001], and 18.30 Ohms on the posterior wall [AUC = 0.77 (0.72–0.83), p<0.0001]. A local impedance drop larger than 21.80 Ohms on the anterior wall and 18.30 Ohms on the posterior wall was associated with an increased probability of effective lesion creation [OR = 11.21, 95%CI 4.22–29.81, p<0.0001; and OR = 7.91, 95%CI 3.77–16.57, p<0.0001, respectively].

**Conclusion:**

The measurement of the local impedance may predict optimal lesion formation. A local impedance drop > 21.80 Ohms on the anterior wall and > 18.30 Ohms on the posterior wall significantly increases the probability of creating a successful lesion.

## Introduction

Pulmonary vein isolation (PVI) is the most effective treatment for paroxysmal atrial fibrillation (AF) [1]. Nonetheless, the recurrence rate and need for a repeated procedure with all its potential consequences are substantial [2]. The development of contact force (CF) sensing ablation catheters resulted in an impressive improvement of the efficacy of radiofrequency (RF) point-by-point PVI procedures [3, 4]. Within the last few years, novel markers have been used for lesion prediction, such as ablation index. The routine use of these parameters resulted in a further increase of procedural success rate [5–7]. However, AF recurrence mostly due to pulmonary vein (PV) reconnection remained an issue [8]; thus, further achievements are warranted to increase the durability of PVI and improve procedural safety by optimizing lesion prediction. The ablation index predicts lesions by incorporating CF, ablation time and power in the formula. Still, it does not take local impedance (LI) change into account, which is a meaningful parameter of lesion formation [7, 9, 10]. Preclinical data are available with a novel CF sensing ablation catheter, which can measure LI changes; however, clinical validation has not been conducted yet [11]. Our preliminary pilot study aimed to evaluate the role of LI drop in lesion prediction during RF ablation for paroxysmal AF with INTELLANAV STABLEPOINT catheter (Boston Scientific, Marlborough, MA, USA) and find an optimal LI drop cut-off value that may predict successful lesion creation.

## Materials and methods

### Procedural workflow

Patients were scheduled for PVI due to symptomatic, drug-refractory paroxysmal AF according to the current guideline [1]. Procedures were performed under conscious sedation. After femoral venous puncture and fluoroscopy-guided double transseptal puncture, a fast anatomical map of the left atrium (LA) was performed with a high-density mapping system (Rhythmia Mapping System, Boston Scientific, Marlborough, MA, USA) and a 64-electrode basket mapping catheter (INTELLAMAP ORION, Boston Scientific, Marlborough, MA, USA). Intravenous unfractionated heparin was administered immediately after the first transseptal puncture to maintain an activated clotting time of >300 seconds.

Point-by-point RF applications were delivered around the antra of the ipsilateral PVs with a 4 mm tip, irrigated, CF sensing ablation catheter (StablePoint catheter), using a steerable sheath (Agilis, S or M curve; Abbott). The basket catheter was placed in the contralateral PVs during ablation to avoid touching it during RF sessions and avoid distortion of ablation data (e.g. CF, LI). Radiofrequency energy was applied in power control mode (40–50 W) with a temperature limit of 43°C. The irrigation rate was 30 ml/min during applications and 2 ml/min during mapping. The ablation points were marked automatically with 6 mm diameter, numbered AutoTags. We applied overlapping ablation points; thus, the inter-tag distances were less than 6 mm between all neighboring points. Predefined AutoTag settings were the following: catheter stability (3 mm for > 3 sec), minimum CF (30% of time > 3 g), and minimal LI drop > 3 Ohms. AutoTag coloring was set based on the LI drop as the following: white <10 Ohm, pink 10–20 Ohm, red >20 Ohm. The LI drop was monitored during the applications to guide the duration of the ablation sessions. We aimed to reach a LI drop of 20–30 Ohms, based on previous experimental data [11].

After completing the circumferential ablation line around the PVs antra, the success of the first-pass RF lesions was evaluated with bipolar pacing at the distal electrode pairs of the ablation catheter along the entire ablation line with an output of 10 mA at 2 ms pulse duration, as described previously [12–15].

Successful RF applications were defined as the inability to capture the tissue at the given ablation site. Unsuccessful lesions were defined as the persistence of the ability to capture the local tissue. AutoTag numbers belonging to successful and unsuccessful lesions were recorded. At unsuccessful sites, additional RF applications were delivered until unexcitability was achieved.

**Fig 1:**
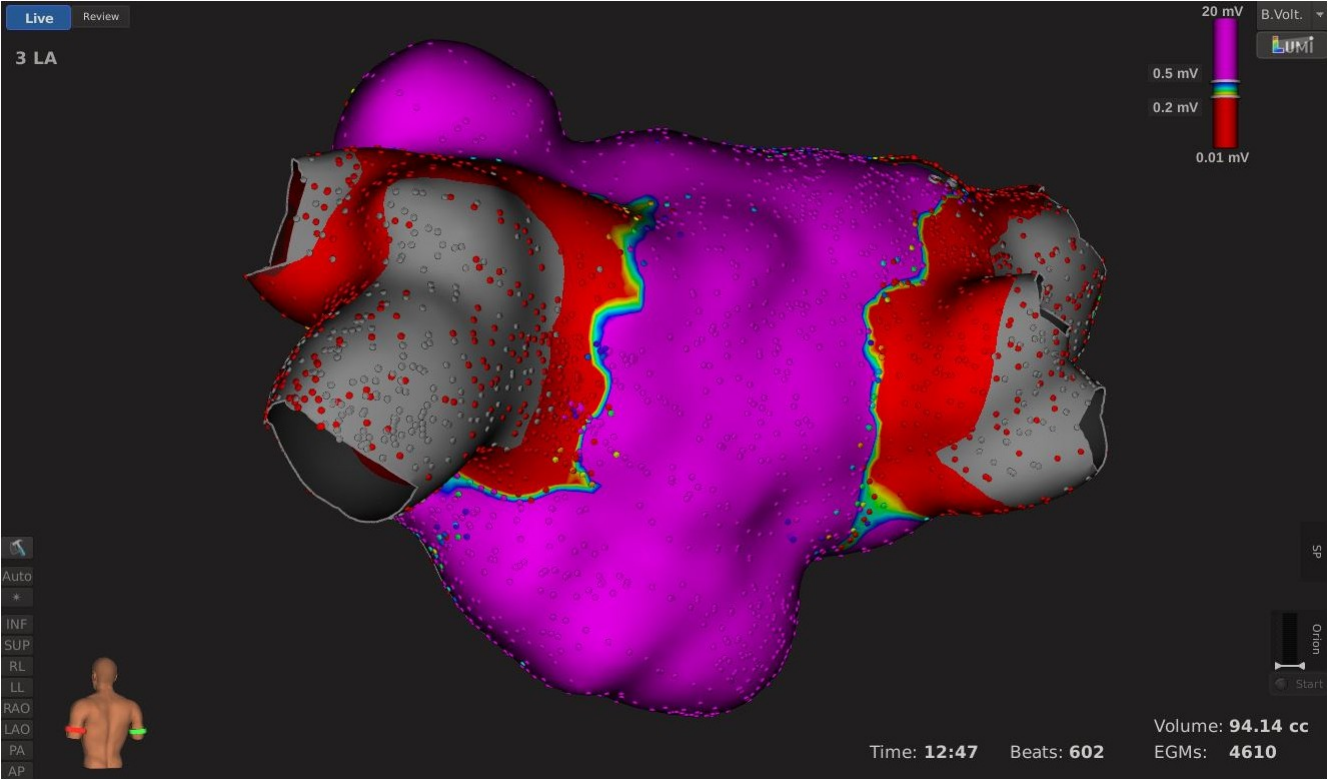
Left atrial bipolar voltage map created with Rhythmia electroanatomical mapping system after successful PVI.

After that, left and right ipsilateral PVs were mapped using the basket catheter, and electrical conduction inside the PVs was analyzed. If LA to PV conduction was present, RF applications targeting conduction gaps on the circumferential line were delivered to complete the PVI. Twenty minutes after the last RF application, entrance and exit block were verified by evaluating the basket catheter’s signals inside all PVs and with pacing maneuvers using the ablation catheter. Finally, a 3-dimensional high-density voltage map of the LA was created to verify the completeness of the isolation line (Fig 1).

### Data collection

The ablation catheter used in the current study is able to measure real-time LI calculated from a local electric field generated at the tip of the catheter. Data of each first-pass ablation point were collected: power, minimum CF, maximum CF, mean CF, duration of application, baseline LI and LI drop. Moreover, the CF range during the applications was calculated by extracting the minimal CF from the maximal CF of the given ablation point. Force time integral (FTI) was calculated by multiplying the mean CF by the application duration. All numbered AutoTag points were exported from the system for off-line analysis.

All patients provided written informed consent to the ablation procedure, data retrieval, and analysis. The study protocol was approved by the Semmelweis University Regional and Institutional Committee of Science and Research Ethics (No: 280/2020) and was in accordance with the Declarations of Helsinki.

### Statistical analysis

The majority of the variables showed non-parametric distributions after performing the Shapiro-Wilk test. The continuous variables were expressed as medians and interquartile ranges, while the categorical variables were expressed as percentages with event numbers. Continuous variables were compared with the Mann-Whitney test. The optimal cut-off values were established using the receiver operating characteristic analyses and were compared by the log-rank test. Univariate logistic regression analysis was performed to determine the predictive value of the LI drop cut-off values. A two-tailed p-value of <0.05 was considered statistically significant. Statistical analyses were performed using IBM SPSS 25 (Apache Software Foundation, USA) and GraphPad Prism 7.1 (GraphPad Softwares Inc., USA), software products.

## Results

### Lesion parameters

**Fig 2:**
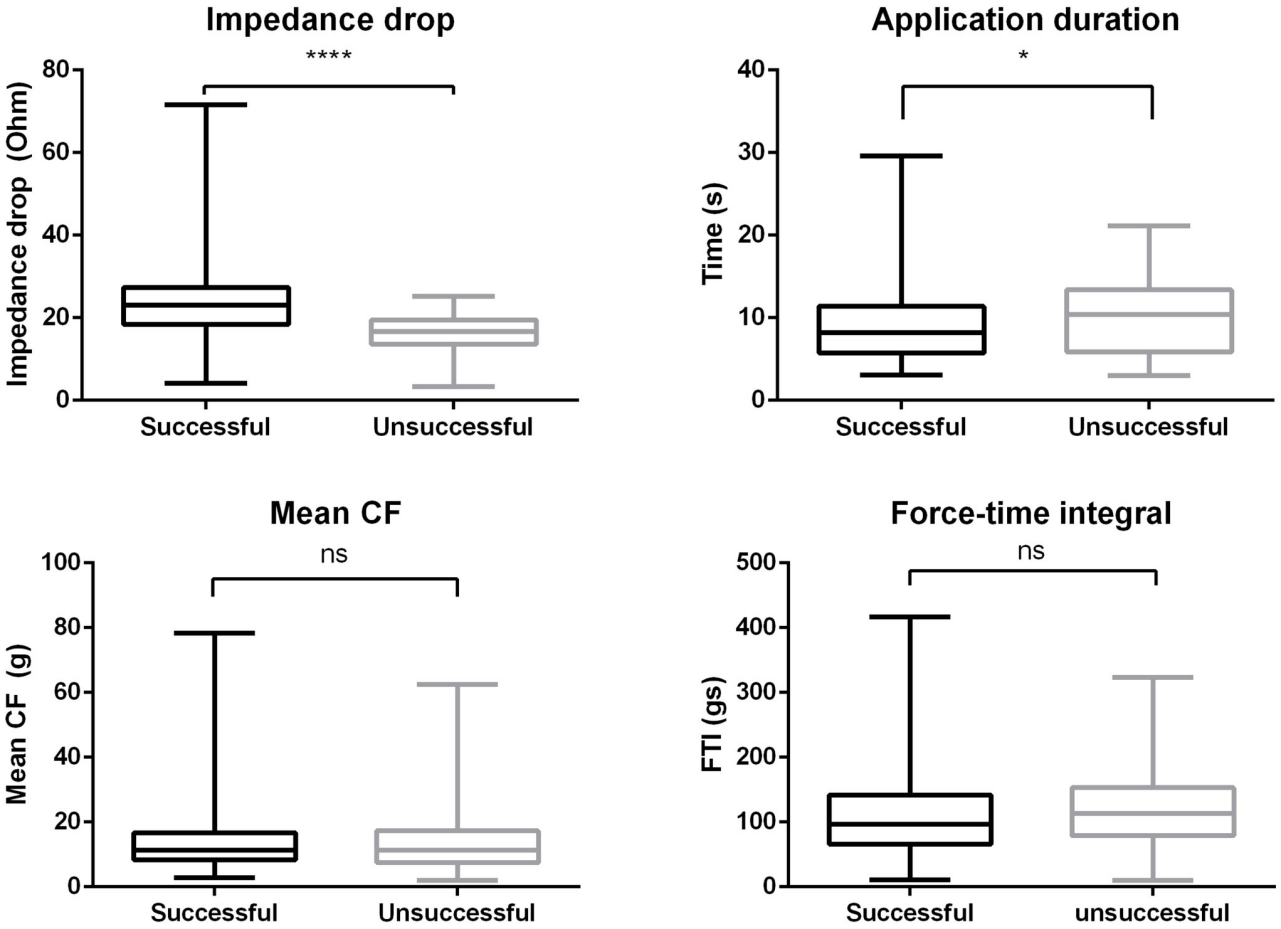
Impedance drop, application duration, mean contact force, and force-time integral parameters of the successful and unsuccessful lesions. Abbreviations: CF = contact force, FTI = force-time integral, * = p<0.05, ****p<0.0001, ns = p≤0.05.

**Fig 3:**
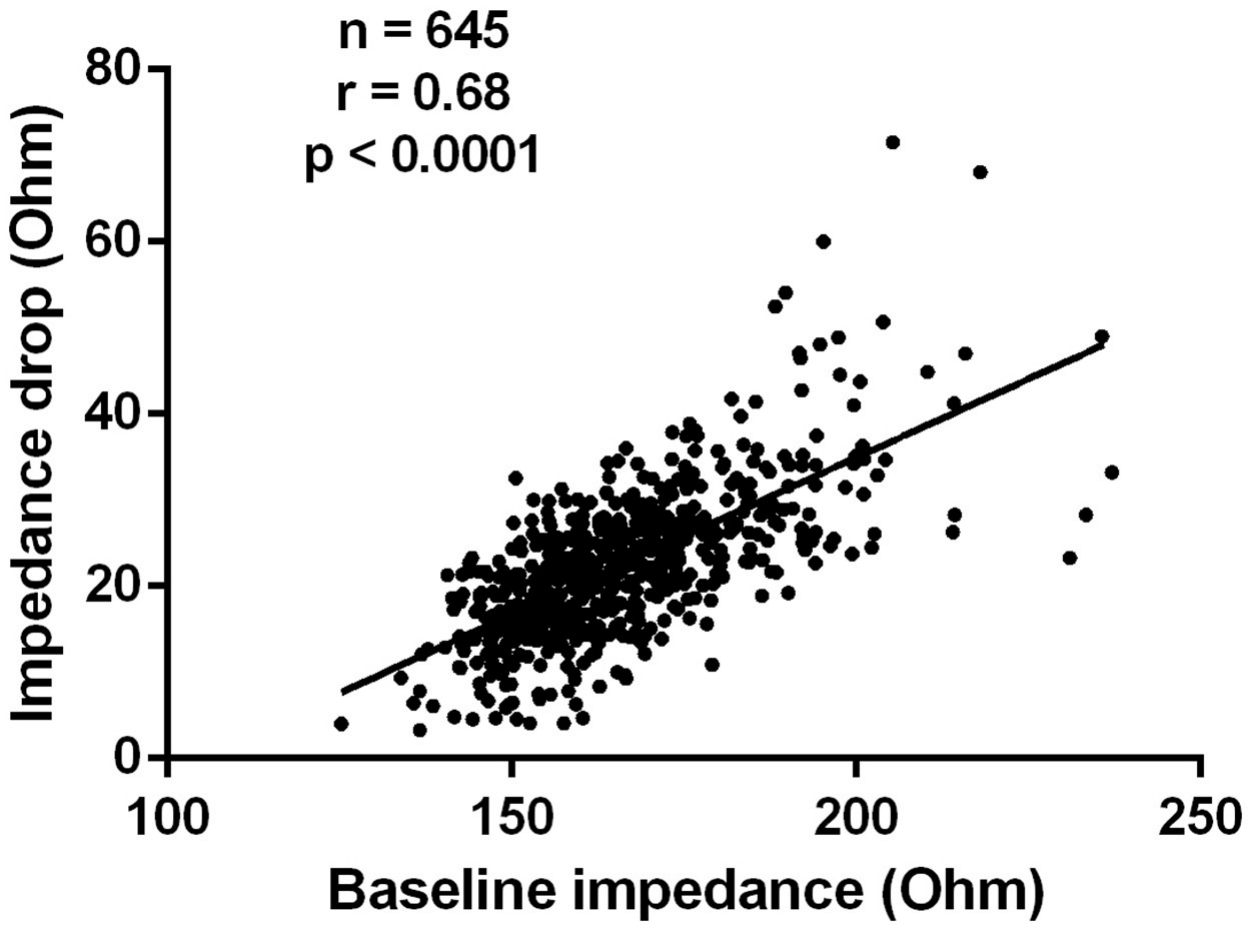
Scatter plot with the line of best fit demonstrating the correlations between starting local impedance and local impedance drop. Abbreviations: n = number of applications, r = correlation coefficient.

Overall, 645 applications of eight subjects were analyzed (baseline characteristics of the study population are shown in Table 1), out of which 561 were successful and 84 were unsuccessful. Compared to the unsuccessful ablation points, the successful applications were shorter (p = 0.0429) and had a larger LI drop (p<0.0001). On the other hand, there was no difference between successful and unsuccessful applications in terms of mean CF (p = 0.8571), minimum CF (p = 0.7093), maximum CF (p = 0.1518), CF range (p = 0.0519), and force-time integral (p = 0.0699). No steam pop occurred. Detailed parameters of the lesions are presented in Table 2 and Fig 2. The LI drop had a significant positive correlation with the baseline LI (Pearson R = 0.68, p < 0.0001), shown in Fig 3. Acute reconnection occurred in one case at the postero-inferior part of the right superior PV after the 20 minutes waiting period. The localization of the unsuccessful applications around the PVs was the following: left anterior 18, left posterior 9, right anterior 16, right posterior 41.

**Table 1:** Baseline characteristics of the study population.

**Patient characteristics (n = 8)**	
Age (years)	53 (43–59)
Sex (male)	8 (100%)
Type of AF	
Paroxysmal	6 (75%)
Persistent	2 (25%
Prior stroke or TIA	0 (0%)
Diabetes	2 (25%)
Hypertension	7 (88%)
Congestive heart failure	1 (13%)
Ischemic cardiomyopathy	1 (13%)
GERD	1 (13%)
CHA_2_DS_2_-VASc score	1 (1–2)
LVEF (%)	65 (55–65)
**Procedure characteristics**	
Procedure time (min)	100 (83–118)
LA dwelling time (min)	90 (67–98)
Absorbed fluoroscopy dose (mGy)	23 (11–26)
DAP (uGym2)	252 (109–302)
Fluoroscopy time (min)	7 (4–11)

Abbreviations: AF = atrial fibrillation; TIA = transient ischemic attack; GERD = gastroesophageal reflux disease; LVEF = left ventricular ejection fraction; DAP = dose area product.

**Table 2:** Characteristics of the successful and unsuccessful lesions.

Parameter	Successful lesions	Unsuccessful lesions	p value
Application duration (s)	8.2 (5.8–11.4)	10.4 (5.9–13.4)	**0.0429**
Baseline impedance	164.7 (156.2–175.1)	155.5 (150.3–161.1)	**<0.0001**
Impedance drop (Ohm)	23.1 (18.4–27.4)	16.7 (13.7–19.5)	**<0.0001**
%LI drop* (%)	13.9 (11.6–16.1)	10.4 (8.6–12.3)	**<0.0001**
Number of applications with 50 W (vs. 40 W)	485 (vs. 76)	68 (vs. 16)	0.1823
Mean CF (g)	11.4 (8.30–16.7)	11.4 (7.6–17.3)	0.8571
Minimum CF (g)	6.0 (3.4–10.0)	6.5 (2.6–11.9)	0.7093
Maximum CF (g)	18.3 (12.5–26.1)	20.6 (13.9–27.4)	0.1518
CF range (g)	10.1 (6.3–17.4)	12.0 (7.4–20.0)	0.0519
FTI (gs)	96.3 (65.8–142.1)	113.0 (78.9–153.2)	0.0699

Abbreviations: CF = contact force, FTI = force-time integral, g = grams, gs = gram*second, LI = local impedance, s = seconds, W = Watts.

* %LI drop was calculated: impedance drop divided by the baseline impedance and multiplied with 100.

### The cut-off value of local impedance drop predicting optimal lesion creation

To establish an optimal cut-point for the LI drop that indicates a successful lesion formation, we used receiver operating characteristic analysis. A LI drop above 21.80 Ohms seemed to be an optimal cut-point for successful lesions on the anterior wall [AUC = 0.80 (0.75–0.86), p<0.0001; sensitivity: 66% (60–71); specificity: 85% (69–95), positive predictive value 98% (95–99), negative predictive value 22% (18–26)]. A LI drop above 18.30 Ohms was an optimal cut-point for successful lesions on the posterior wall [AUC = 0.77 (0.72–0.83), p<0.0001; sensitivity: 66% (60–72); specificity: 80% (66–90), positive predictive value 94% (91–97), negative predictive value 32% (27–37)].

**Fig 4:**
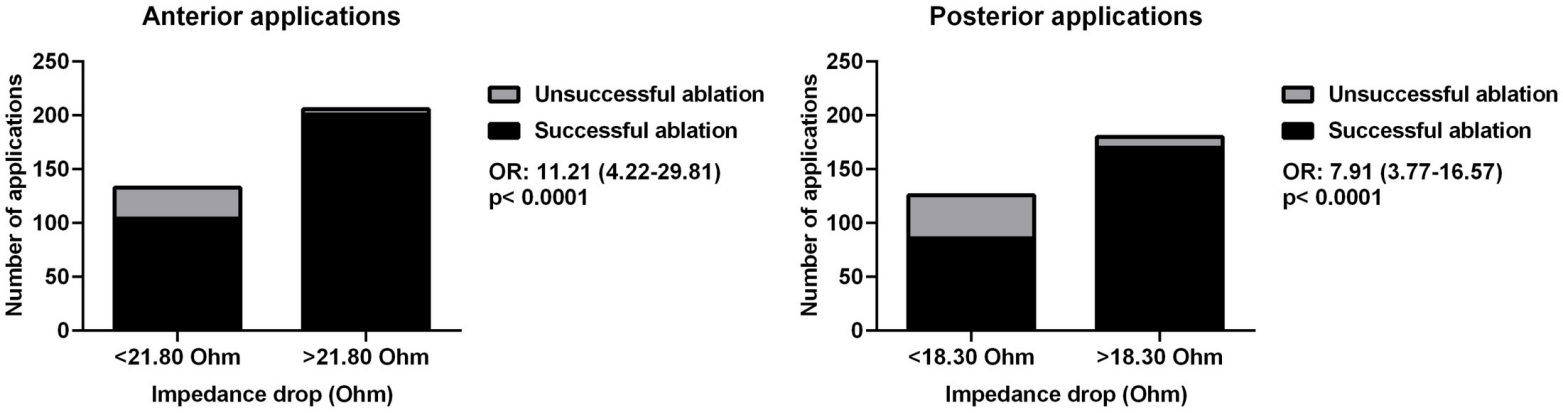
Logistic regression analysis of lesion efficacy on the anterior (left side) and posterior (right side) walls. Abbreviation: OR = odds ratio.

Next, we created groups of patients with LI drop below and over the previously established cut-points. We analyzed the predictive value of the LI drop on lesion efficacy with logistic regression analysis. A LI drop larger than 21.80 Ohms was significantly associated with an increased probability of effective lesion creation on the anterior wall [OR = 11.21, 95%CI 4.22–29.81, p<0.0001]. A LI drop larger than 18.30 Ohms was significantly associated with an increased probability of effective lesion creation on the posterior wall [OR = 7.91, 95%CI 3.77–16.57, p<0.0001]. These results are shown in Table 3 and Fig 4.

**Table 3:** The number of successful and unsuccessful lesions used for sensitivity, specificity and predictive value calculation. Applications below and above the LI cut-points are shown separately to enable the identification of true positive and negative, false positive and negative values.

Anterior wall	Successful lesions	Unsuccessful lesions
LI drop > 21.80 Ohms	201	5
LI drop < 21.80 Ohms	104	29
Posterior wall	Successful lesions	Unsuccessful lesions
LI drop > 18.30 Ohms	170	10
LI drop < 18.30 Ohms	86	40

Abbreviations: LI = local impedance.

## Discussion

### Main findings

The current study’s main finding is that local impedance measurement predicts successful lesion formation, and a local impedance drop > 21.80 Ohms on the anterior wall and > 18.30 Ohms on the posterior wall significantly increases the probability of having a successful lesion.

### Evaluation of lesion efficacy with “loss of capture” technique

The efficacy of lesion formation was evaluated by the loss of local capture in the current study. This technique was widely studied previously, and it was found to be a good indicator of durable lesion creation. Steven et al. showed that pace capture along the ablation line could be used to identify conduction gaps [15]. Similarly, pacing for unexcitability during PVI was found to safely identify potential sites of dormant conduction in two other studies [13, 14]. Moreover, the endpoint of loss of capture along the PVI line was shown to improve the 5-year success rate [12].

### High power ablation and lesion creation in the LA

We showed that ablations with higher power were effective in the LA, which is not surprising based on the results of previous experimental studies [16, 17]. Bourier et al. found that high power short duration ablation results in a different lesion geometry (e.g. larger diameters but smaller depth) compared to conventional lower power ablation. Still, the depth of the high-power applications is sufficient to reach transmural lesions in the atria [17]. Moreover, the larger diameters might improve the chance of creating a contiguous lesion set.

### The relevance of the local impedance drop measurement in the lesion prediction

The measurement of LI is more accurate to guide ablations compared with the generator impedance [18]. Clinical data on the usefulness and optimal cut-off LI drop are available in case of the IntellaNav MiFi OI (Boston Scientific, Marlborough, MA, USA) catheter [19, 20]. Our results are similar to the LOCALIZE clinical trial, as we showed that LI drop is predictive for lesion creation, a higher starting LI is correlated with a larger LI drop, and different LI drop cut-off values should be used on the anterior and posterior wall. There are some differences between the results, arising from the following facts. First, the device used in the LOCALIZE trial was an ablation catheter, which generates an electrical field between one of the electrodes 1 and 4, and uses microelectrodes embedded in the catheter tip and electrode 2 for LI measurement [19]. However, the currently investigated IntellaNav StablePoint catheter does not have micro-electrodes and it measures the LI between the distal electrode pair (electrode 1 and 2). Thus, the LI values are different and data derived from the IntellaNav Mifi OI catheter evaluation cannot be used in the case of the StablePoint catheter. We found higher absolute values of LI in the current study compared to the LI values of the LOCALIZE trial. Second, ablations were performed with a non-CF sensing catheter using a 25–40 W power setting in the LOCALIZE trial, and the inter-lesion distance was ≤ 6 mm in only 53% of segments. These facts might impair the probability of achieving a contiguous lesion set, which is confirmed by the 16.8% acute reconnection rate, which is higher than the acute reconnection rate of ablation with CF sensing catheters [5]. The current study was performed with a 40–50 W power setting and a contact force-sensing ablation catheter. Overall, a better lesion quality is more likely using a CF sensing catheter and high power ablations, resulting in more contiguous, more uniform, and transmural lesions. We experienced acute reconnection only in one of the ablated PVs (3.1%) in our current study. Third, the LOCALIZE study included a larger number of points in the analysis; however, only mean LI parameters per segment were considered in the statistical analysis. Regional differences (e.g. anterior and posterior LI drop cut-points) were calculated from those segments that had an inter-lesion distance ≤ 6 mm; thus, the final analysis was performed with 416 LI data [19]. Our study evaluated LI parameters per ablation point in a one-by-one manner, resulting in 645 LI data.

Preclinical data with the new catheter were presented by Garrott et al., who showed that LI drop correlates with the lesion depth both in vitro and in vivo. Thus, LI drop provides information regarding sufficient lesion creation (LI drop >20 Ohms), and it also indicates excessive heating that might lead to an increased risk of steam pop (impedance drop >65 Ohms). They found that the total RF time was significantly reduced when using LI guidance. Moreover, a variation in baseline LI was reported based on catheter inclination [11]. Until now, no clinical study was performed to evaluate the optimal LI drop value for effective lesion formation with the StablePoint catheter. Our current findings showed that LI measurement predicts successful lesion formation, and we established the optimal LI drop cut-off values both on the anterior and posterior wall that indicate a successful lesion creation. Moreover, we also showed that the duration of RF applications could be significantly shorter when using LI guidance.

### Clinical utility of the current findings

The current study has potential clinical consequences. We showed that the LI drop provides excellent feedback regarding optimal lesion creation. The currently presented cut-points showed an impressive sensitivity, specificity, negative and positive predictive value. We found no difference in mean, minimum, maximum CF, CF range, and FTI between successful and unsuccessful applications when using LI drop guidance. It is likely that catheter stability is essential, and applications with unstable catheter positions will lead to a less effective lesion creation, despite an optimal mean CF value.

Application time was shorter in case of successful ablation points in the present study. This might be useful to prevent "overshooting" and having potentially very large impedance drops; thus, reducing the risk of steam pops. Moreover, RF applications should be short as possible in case of high power ablations—especially on the posterior wall—to avoid potentially severe consequences (e.g. esophageal lesions); thereby, LI guidance might add to the safety of the procedure [21–23]. Based on our data, a LI drop > 22 Ohms on the anterior wall and > 18 Ohms on the posterior wall might be valuable cut-points indicating an optimal lesion creation during PVI in clinical practice. Indeed, further clinical studies are necessary to verify these results on larger patient populations with long-term follow-up.

### Limitations

Our pilot study has limitations. The number of included patients is not very large; on the other hand, the number of analyzed lesions was big enough to reach a clear statistical significance. Moreover, follow-up data are missing that would be necessary to evaluate the clinical value of the findings; thus, further clinical investigation will be necessary in the future. We also mention the fact that LI measurements are currently only possible with the one proprietary catheter and the connected electrophysiology system, both owned by the same company. This system is not able to display any other advanced metric (that would incorporate application power) of lesion creation.

## Conclusions

The measurement of LI may predict optimal lesion formation. A LI drop > 21.80 Ohms on the anterior wall and > 18.30 Ohms on the posterior wall significantly increases the probability of having a successful lesion.

## Supporting information

S1 File (DOCX)
