# Supplementary material for: The role of local impedance drop in the acute lesion efficacy during pulmonary vein isolation performed with a new contact force sensing catheter—A pilot study
Source: PLoS One. 2021 Sep 16;16(9):e0257050. doi: 10.1371/journal.pone.0257050 (PMC8445471; doi:10.1371/journal.pone.0257050)
Supplement: S1 File — (DOCX) [file pone.0257050.s001.docx]

#### Your choices:

Test chosen: Power of a "not significant" unpaired *t* test

|  | N | SD |
| --- | --- | --- |
| Group 1 | 561 | 8.43 |
| Group 2 | 84 | 5.087 |

Significance level (alpha) = 0.05 (two-tailed)

**Explanation for 90% power:**

Assume that the true difference between means is 3.07. Now imagine that you perform many experiments, with the same sample size used in the completed experiment. Due to random sampling, you won't find that the difference between means equals 3.07 in every experiment. Instead, you'll find that the difference between means will be greater than 3.07 in about half the experiments, and less than 3.07 in the other half.

In 90% (the power) of those experiments, the P value will be less than 0.05 (two-tailed) so the results will be deemed "statistically significant". In the remaining 10% of the experiments, the P value will be greater than 0.05 (two-tailed) so the results will be deemed "not statistically significant" and you will have made a Type II (beta) error.

Summary: Your experiment had a 90% power to detect a difference between means of 3.07 with a significance level (alpha) of 0.05 (two-tailed).

**Table of tradeoffs:**

For any power you choose, this table shows the difference between means that can be detected.

| **Delta** |  | **Power (%)** |
| --- | --- | --- |
| 4.07 |  | 99 |
| 3.42 |  | 95 |
| 3.07 |  | **90** |
| 2.84 |  | 85 |
| 2.66 |  | 80 |
| 2.50 |  | 75 |
| 2.36 |  | 70 |
| 2.10 |  | 60 |
| 1.86 |  | 50 |
| 1.62 |  | 40 |
| 1.36 |  | 30 |
| 1.06 |  | 20 |
| 0.64 |  | 10 |

Report created by GraphPad StatMate 2.00.   2021. 06. 28. 11:36:57
